# Enhanced Active Targeting via Cooperative Binding of Ligands on Liposomes to Target Receptors

**DOI:** 10.1371/journal.pone.0067550

**Published:** 2013-06-28

**Authors:** Tomoki Sugiyama, Tomohiro Asai, Yuki Murase Nedachi, Yasufumi Katanasaka, Kosuke Shimizu, Noriyuki Maeda, Naoto Oku

**Affiliations:** 1 Department of Medical Biochemistry, School of Pharmaceutical Sciences, University of Shizuoka, 52-1 Yada, Suruga-ku, Shizuoka, Japan; 2 Nippon Fine Chemical Co. Ltd., Takasago, Hyogo, Japan; Okayama University, Japan

## Abstract

To achieve effective active targeting in a drug delivery system, we previously developed dual-targeting (DT) liposomes decorated with both vascular endothelial growth factor receptor-1 (VEGFR-1)-targeted APRPG and CD13-targeted GNGRG peptide ligands for tumor neovessels, and observed the enhanced suppression of tumor growth in Colon26 NL-17 tumor-bearing mice by the treatment with the DT liposomes encapsulating doxorubicin. In this present study, we examined the binding characteristics of DT liposomes having a different couple of ligands, namely, APRPG and integrin α_v_β_3_-targeted GRGDS peptides. These DT liposomes synergistically associated to stimulated human umbilical vein endothelial cells compared with single-targeting (ST) liposomes decorated with APRPG or GRGDS. The results of a surface plasmon resonance assay showed that ST liposomes modified with APRPG or GRGDS peptide selectively bound to immobilized VEGFR-1 or integrin α_v_β_3_, respectively. DT liposomes showed a higher affinity for a mixture of VEGFR-1 and integrin α_v_β_3_ compared with ST liposomes, suggesting the cooperative binding of these 2 kinds of ligand on the liposomal surface. In a biodistribution assay, the DT liposomes accumulated to a significantly greater extent in the tumors of Colon26 NL-17 tumor-bearing mice compared with other liposomes. Moreover, the intratumoral distribution of the liposomes examined by confocal microscopy suggested that the DT liposomes targeted not only angiogenic endothelial cells but also tumor cells due to GRGDS-decoration. These findings suggest that "dual-targeting" augmented the affinity of the liposomes for the target cells and would thus be useful for active-targeting drug delivery for cancer treatment.

## Introduction

Polyethylene glycol (PEG)-modified liposomes are well known to have the characteristic of long circulation, to accumulate in inflammatory site or in the interstitial space of tumor tissues owing to the enhanced permeability and retention (EPR) effect [1], [2]. In fact, PEG-liposomes containing doxorubicin (DOX), trademarked as Doxil, have been clinically used for cancer treatment. Although PEG-liposomes are useful for passive targeting to such tissues having a leaky endothelium, PEGylation is also known to suppress the interaction of liposomes with target cells [3], [4]. For overcoming this so-called PEG dilemma, active targeting of liposomes has been widely investigated by modifying liposomes with ligands such as antibodies and peptides as active-targeting probes [5]-[7]. In the case of tumor targeting, the ligands are usually conjugated to the end of the PEG chain [8], [9].

We previously isolated a cancer neovessel-specific peptide, Ala-Pro-Arg-Pro-Gly (APRPG containing the PRP motif) [10], [11], which selectively binds to vascular endothelial growth factor receptor-1 (VEGFR-1) [12]. Similarly, Ricardo revealed that CPQPRPLC phage of a phage-displayed library bind specifically to VEGFR-1 [13]; and Arap and coworkers identified Asn-Gly-Arg (NGR) and Arg-Gly-Asp (RGD) motifs [14], which specifically bind to CD13 (aminopeptidase N) and integrin α_v_β_3_, respectively [15], [16]. Peptides such as APRPG, GNGRG, and GRGDS are useful as targeting probes of liposomes since modification of liposomes with any one of these peptides enhances the anticancer activity of DOX encapsulated in such liposomes in tumor-bearing mice [17], [18].

To enhance the ability of a liposomal drug carrier to actively target tumor neovessels, we previously proposed a new DDS technology, namely dual-targeting (DT), in which each liposome is decorated with 2 different ligands [19]. We found that DT liposomes decorated with both APRPG and GNGRG bind to a significantly greater extent to human umbilical vein endothelial cells (HUVECs) compared with single-targeting (ST) liposomes and, also, that they afford greater suppression of tumor growth in Colon26 NL-17 tumor-bearing mice injected with DOX-encapsulating DT liposomes.

In the present study, we used a different couple of ligands, i.e., APRPG and GRGDS, for clarifying the usefulness of this DT strategy. Moreover, this couple of ligands targets not only angiogenic endothelial cells but also cancer cells, because the target molecule of GRGDS, i.e., integrin α_v_β_3,_ is expressed on both kinds of cells. We analyzed the intermolecular interaction between DT liposomes and target molecules to investigate the mechanism of dual-targeting. For this purpose, we used biosensor technology based on surface plasmon resonance (SPR), which is accepted as a standard technique in biochemistry and other related sciences because it can give reliable kinetic data on the interaction of various molecular partners. During recent years, therefore, SPR has been used for monitoring the membrane binding of peptides and of peripheral proteins participating in membrane-mediated cell signaling. We studied the interaction between DT liposomes and their target molecules (VEGFR-1 and α_v_β_3_ integrin).

## Materials and Methods

### Preparation of Liposomes

Distearoylphosphatidylcholine (DSPC), methoxy PEG2000-distearylphosphatidyl- ethanolamine (DSPE-PEG), DSPE-PEG-APRPG, DSPE-PEG-GRGDS, and cholesterol were the products of Nippon Fine Chemical, Co. Ltd (Takasago, Hyogo, Japan). DSPC and cholesterol with DSPE-PEG, DSPE-PEG-APRPG or DSPE-PEG-GRGDS (10∶5:1 as a molar ratio), or with DSPE-PEG-APRPG and DSPE-PEG-GRGDS (10∶5:0.5∶0.5 as a molar ratio) were dissolved in chloroform, dried under reduced pressure, and stored *in vacuo* for at least 1 h. Then the resulting thin lipid film was hydrated with HEPES-buffered saline (HBS, pH 7.4) and frozen and thawed for 3 cycles by using liquid nitrogen to form liposomes. PEG-decorated liposomes (PEG-Lip), APRPG-PEG-decorated liposomes (PRP-PEG-Lip), GRGDS-PEG-decorated liposomes (RGD-PEG-Lip), and PEG liposomes decorated with both APRPG and GRGDS (Dual-PEG-Lip) were sized by extruding them 5 times through a polycarbonate membrane filter with 100-nm-pores (Nucleopore, Maidstone, UK). Particle size and ζ-potential of the liposomes diluted with HBS were measured by use of a Zetasizer Nano ZS (MALVERN, Worcestershire UK, USA).

For determining the association of liposomes with HUVECs and observing the intratumoral distribution of liposomes, we added 1,1′-dioctadecyl-3,3,3′,3′-tetramethyl-indocarbocyanine perchlorate (DiIC_18_, Molecular Probes Inc., Eugene, OR, USA) to the initial chloroform solution at a dose of 5 mol% of DSPC. In the case of the biodistribution assay, a trace amount of [^3^H]cholesteryl hexadecyl ether (GE Healthcare UK Ltd., Buckinghamshire, England) was added to the initial solution.

### Liposomal Association with HUVECs

HUVECs (Lonza, Walkersville, MD, USA) were seeded into gelatin-coated 24-well plates (2×10^4^ cells/well) and cultured in endothelial cell growth medium-2 (EGM-2, Lonza) at 37°C for 48 h in a humidified atmosphere of 5% CO_2_ in the air. Then, DiIC_18_-labeled liposomes were added (final concentration of 0.05 or 0.1 mM as DSPC concentration), and the cultures were incubated for 4 h at 37°C. Next, after these cells had been washed with ice-cold PBS, they were solubilized in 0.1% sodium dodecylsulfate-containing 10 mM Tris-HCl, pH 7.4. The amount of DiIC_18_-labeled liposomes associated with the HUVECs was fluorometrically determined at an excitation wavelength of 549 nm and an emission wavelength of 592 nm by use of an Infinite M200 (Tecan, Grödig, Austria). The amount of proteins in the samples was determined by performing the BCA protein assay (Pierce Chemical, IL). The amount of liposomes associated with the HUVECs was corrected by the amount of cellular proteins.

### Surface Plasmon Resonance Binding Assay

The SPR binding assay was performed with a Biacore 2000 system (GE Healthcare, Facility of IFR 128 Gerland, Lyon Sud, France) to characterize the interactions of the peptides presented on the liposomal surface with VEGFR-1 and/or integrin α_v_β_3_. The Biacore sensor chip CM5 (GE Healthcare, Buckinghamshire, England, UK) was activated with an Amine Coupling Kit (GE Healthcare) based on 1-ethyl-3-(3-dimethylaminopropyl)carbodiimide·HCl and *N*-hydroxysuccinimide, and coated with recombinant human sVEGFR-1 (Flt-1, 2 µg/100 µL in acetate buffer, pH 4.5, PromoKine, Heidelberg, Germany) and/or integrin α_v_β_3_ (15 µg/100 µL, R&D Systems, Minneapolis, MN, USA). Ethanolamine was used as a blank. Liposomes (1 mM as DSPC) dissolved in HBS buffer containing EDTA, NaCl, and surfactant P20 were applied to the sensor chip for binding analysis using the Biacore system (injection time, 3 min; flow rate, 20 µl/min).

### Biodistribution Study

Colon 26 NL-17 cells, a murine colon adenocarcinoma 26 subline with high metastatic potential, were established by Dr. Takao Yamori (Japanese Foundation for Cancer Research, Tokyo, Japan) [20] and kindly gifted by Dr. Nakajima (SBI Pharmaceuticals, Tokyo, Japan). Colon 26 NL-17 cells were cultured in DME/Ham’s F12 medium (WAKO, Osaka, Japan) supplemented with streptomycin (100 µg/mL), penicillin (100 units/mL), and 10% fetal bovine serum (FBS) at 37°C in 5% CO_2_. For preparation of Colon26 NL-17 tumor-bearing mice, 1.0×10^6^ cells were implanted subcutaneously into the posterior flank of 4-week-old BALB/c male mice (Japan SLC Inc., Shizuoka, Japan). The biodistribution study was performed at day 10 after tumor implantation. Size-matched Colon26 NL-17-bearing mice were injected with the radiolabeled liposomes via a tail vein (74 kBq/mouse). Twenty-four hours after the injection, the mice were sacrificed under deep anesthesia for collection of the blood. The plasma was obtained by centrifugation (600 *g* for 5 min). Then the heart, lungs, liver, spleen, kidneys, and tumor were removed, washed with saline, and weighed. The radioactivity in each organ was determined with a liquid scintillation counter (Aloka LSC-3100). Distribution data were presented as % dose per 100 mg tissue. The total amount in the plasma was calculated based on the body weight of mice, where the plasma volume was assumed to be 4.27% of the body weight based on the data of total blood volume. The animals were cared for according to the animal facility guidelines of the University of Shizuoka. All animal procedures were approved by the Animal and Ethics Review Committee of the University of Shizuoka.

### Intratumoral Localization of Liposomes

Colon26 NL-17 cells (1.0×10^6^ cells/mouse) were inoculated as described above. Ten days after the tumor implantation, DiIC_18_-labeled liposomes were administered via a tail vein of the mice. The mice were sacrificed under deep anesthesia at 3 h after the liposomal injection, and the tumors were dissected; and the tumor tissues were subsequently embedded in optimal cutting temperature compound (Sakura Finetech, Tokyo, Japan) and frozen at −80°C. Tumor sections (10 µm) were prepared with a cryostat microtome (HM 505E, Microm, Walldorf, Germany), mounted on MAS-coated slides (Matsunami Glass Ind., Japan), air-dried for 1 h, and washed twice with PBS. Endogenous avidin activity was blocked with a blocking reagent kit (Vector Laboratories, CA, USA). After these sections had been blocked with 1% BSA in PBS, they were incubated with biotinylated anti-mouse CD31 rat monoclonal antibody (Becton Dickinson Lab., Franklin Lakes, NJ, USA) for 18 h at 4°C and then visualized after incubation with streptavidin-Alexa fluor 488 conjugates (Molecular Probes Inc., Eugene, OR, USA) for 30 min at room temperature in a humid chamber. The sections were mounted with Perma Fluor Aqueous Mounting Medium (Thermo Shandon, PA, USA), and the fluorescence was observed with a confocal laser-scanning microscope, LSM 510 META (Carl Zeiss, Co. Ltd., Germany).

### Statistical Analysis

Differences in a group were evaluated by an analysis of variance (ANOVA) with the Tukey *post-hoc* test.

## Results

### Association of Dual-targeting Liposomes with HUVECs or their Target Molecules

**Figure 1:**
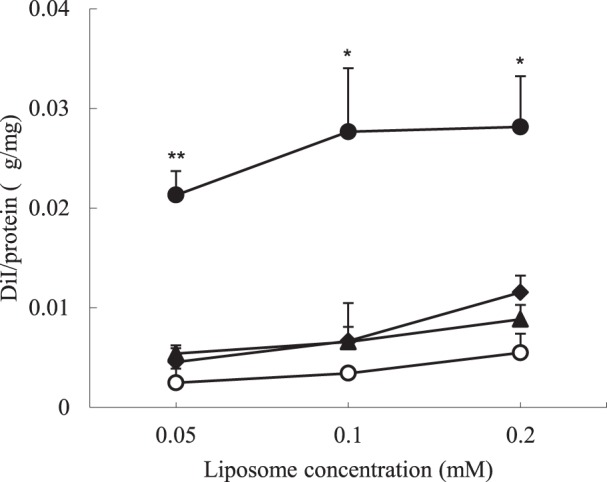
Association of ST and DT liposomes with HUVECs. HUVECs (2×10^4^ cells) cultured for 48 h were incubated in the presence of DiIC_18_-labeled PEG-Lip (○), PRP-PEG-Lip (▴), RGD-PEG-Lip (♦) or Dual-PEG-Lip (•) for 4 h at 37°C. After washing, the amount of liposomes associated with the HUVECs was determined fluorometrically. Association of liposomes is presented as the amount of DiIC_18_ per cellular protein amount. Data are presented as the mean value and SD. Significant differences: *, *P*<0.05; **, *P*<0.01.

At first, the characteristics of the various liposomes were determined. All of the kinds of liposomes used in the present study were approx. 120 nm in diameter and had a ζ-potential of about −6 mV (Table 1). Next, the association of ST and DT liposomes with HUVECs was determined fluorometrically by use of DiIC_18_-labeled liposomes. As shown in Figure 1, ST liposomes, i.e., PRP-PEG-Lip and RGD-PEG-Lip, were highly associated (bound and/or taken up) with the HUVECs compared with PEG-Lip in a dose-dependent manner. The association between Dual-PEG-Lip and HUVECs was significantly higher than that of PEG-Lip or ST liposomes and the cells. Moreover, the association of these Dual-PEG-Lip with the HUVECs seemed to be not additive but synergistic.

**Table 1:** Characteristics of the liposomes examined.

	Liposome-decoration			Particle size	ζ-potential
	(Molar ratio to DSPC)			(nm)	(mV)
	PEG	APRPG	GRGDS		
PEG-Lip	0.1	–	–	119.3±11.0	−5.4±1.4
PRP-PEG-Lip	–	0.1	–	114.7±9.8	−6.5±0.8
RGD-PEG-Lip	–	–	0.1	123.0±7.4	−6.9±1.5
Dual-PEG-Lip	–	0.05	0.05	122.7±8.8	−6.4±4.2

**Figure 2:**
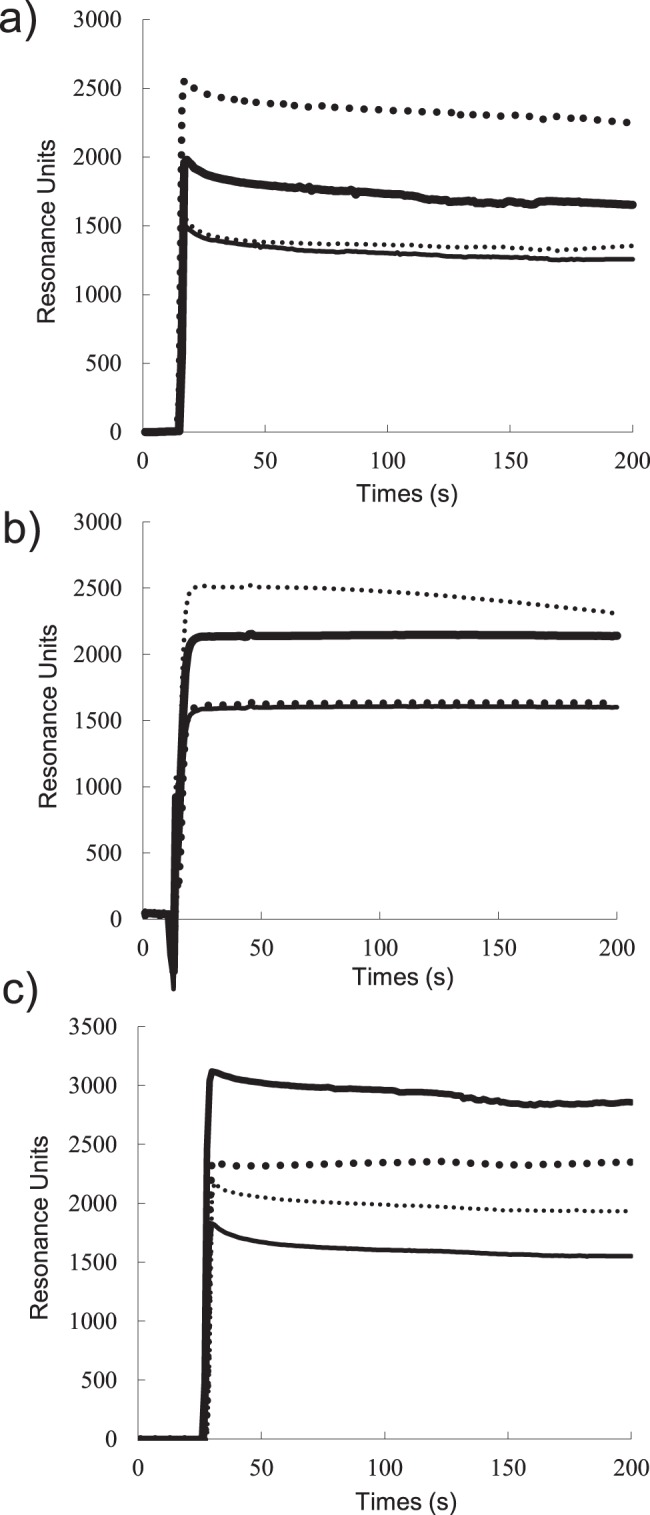
Binding of ST and DT liposomes to the immobilized target molecules of the liposomal ligands. Liposomes (1 mM as DSPC) dissolved in HBS, pH 7.4, containing surfactant P20 were applied to a Biacore sensor chip, CM5, pre-coated with recombinant human VEGFR-1 (a), integrin α_v_β_3_ (b) or both VEGFR-1 and integrin α_v_β_3_ (c) for 3 min at a flow rate of 20 µl/min. Binding of PEG-Lip (thin solid lines), PRP-PEG-Lip (thick dotted lines), RGD-PEG-Lip (thin dotted lines),or Dual-PEG-Lip (thick solid lines) was evaluated by SPR.

To elucidate this cooperative effect of these 2 ligands for the association of Dual-Lip with the cells, we next used the Biacore system to determine the ability of Dual-Lip to bind to immobilized target molecules, namely, VEGFR-1 and integrin α_v_β_3_. PRP-PEG-Lip and Dual-PEG-Lip showed specific binding to VEGFR-1. In contrast, the binding ability of RGD-PEG-Lip toward VEGFR-1 was similar to that of PEG-Lip (Fig. 2a). We assumed that the resonance units with PEG-Lip, as the control, indicated non-specific binding. On the contrary, RGD-PEG-Lip and Dual-Lip showed specific binding to integrin α_v_β_3_, and the resonance units for PRP-PEG-Lip were almost the same as those for the control PEG-Lip (Fig. 2b). Interestingly, as shown in Figure 1c, DT liposomes (Dual-PEG-Lip) showed high affinity for a mixture of VEGFR-1 and integrin α_v_β_3_ compared with ST liposomes (PRP-PEG-Lip or RGD-PEG-Lip), although the total number of ligand molecules per liposome was the same between DT and ST liposomes.

### Biodistribution and Intratumoral Distribution of DT Liposomes

**Figure 3:**
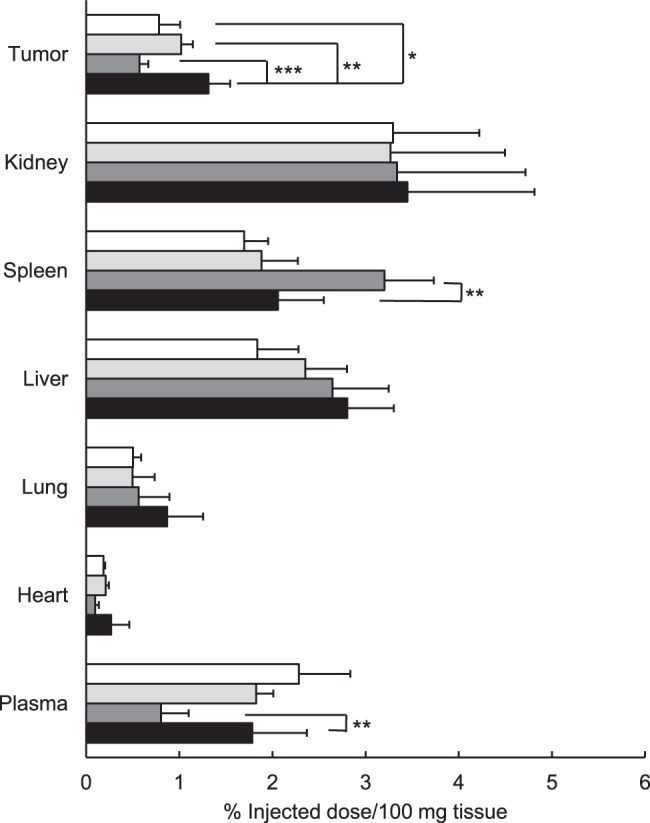
Biodistribution of ST and DT liposomes in tumor-bearing mice. Radiolabeled PEG-Lip (open bar), PRP-PEG-Lip (light grey bar), RGD-PEG-Lip (dark grey bar), or Dual-PEG-Lip (closed bar) were injected into Colon26 NL-17-bearing mice (*n* = 5) via a tail vein at 10 days after the tumor inoculation. At 24 h after the injection, the radioactivity in each organ was determined. Data are shown as a percent of the injected dose per 100 mg tissue and SD. Significant differences: *, *P*<0.05; **, *P*<0.01; ***, *P*<0.001.

The biodistribution of the liposomes at 3 h after an i.v. injection of liposomes into Colon26 NL-17 carcinoma-bearing mice is shown in Figure 3. Plasma concentration of RGD-PEG-Lip was lower than that of the other liposomes, but these liposomes showed the highest accumulation in the spleen. Dual-PEG-Lip did not accumulate in the spleen, even though they contained half of the amount of GRGDS peptides in comparison to RGD-PEG-Lip. Moreover, DT liposomes maintained the characteristic of long circulation like the PEG-Lip, indicating that ligand decoration did not impair this characteristic obtained by PEGylation. Importantly, the DT liposomes accumulated in the tumor to a significantly higher extent than the other liposomal formulations tested.

**Figure 4:**
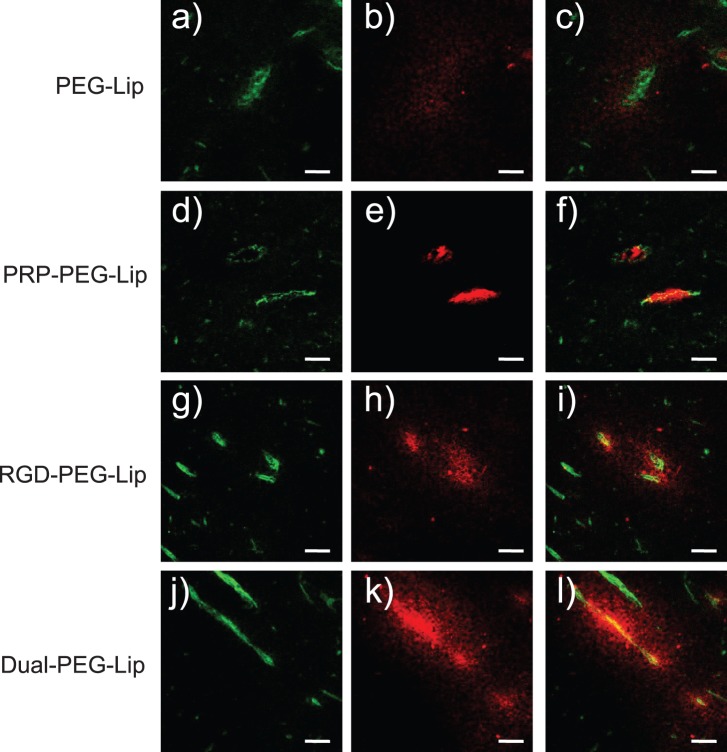
Intratumoral distribution of ST and DT liposomes. DiI fluorescence-labeled PEG-Lip (a–c), PRP-PEG-Lip (d–f), RGD-PEG-Lip (g–i) or Dual-PEG-Lip (j–l) were intravenously injected into Colon26 NL-17-bearing mice at day 10 after tumor implantation. At 3 h after injection, the tumors were dissected, and then frozen-sections (10-µm thickness) were prepared. Left panels (a, d, g, and j) show the distribution of endothelial cells as visualized by immunostained CD31 (green color); and middle panels (b, e, h, and k), the distribution of the liposomes (red color). Merged images are shown in the right panels (c, f, i, and l). Scale bars represent 20 µm.

Finally, the intratumoral localization of the liposomes was determined to evaluate the affinity of Dual-PEG-Lip for angiogenic vessels and tumor cells. As shown in Figure 4, PEG-Lip was observed around tumor vessels, indicating that they had extravasated from these vessels and stayed there due to the EPR effect. In contrast, PRP-PEG-Lip became localized on angiogenic vessels, indicating specific interaction of APRPG with VEGFR on angiogenic endothelial cells. In the case of RGD-PEG-Lip, these liposomes accumulated mostly in the area peripheral to angiogenic vessels; but some of them were detected on angiogenic vessels. According to the interaction of either ligand with its target molecule, Dual-PEG-Lip were localized on angiogenic vessels and in areas peripheral to them.

## Discussion

Angiogenesis is a critical event for both the growth and maintenance of tumors [21]. We originally proposed antineovascular therapy (ANET), which causes indirect tumor regression through damaging angiogenic vessels by the delivery of anticancer drugs to tumor neovessels via liposomal DDS [10]. ANET is effective against drug-resistant tumors [22] and hypovascular pancreatic tumor models [23]. ANET is expected to have a broad anticancer spectrum, since angiogenic endothelial cells are derived from the same progenitor cells in spite of the difference in tumor type. Moreover, the therapeutic effect of ANET would be higher than that obtained by targeting cancer cells, since a large number of cancer cells are maintained by a relatively smaller number of endothelial cells for their growth and maintenance [24]. However, ANET has not yet been shown to be sufficiently effective in therapeutic experiments. Thus, more effective active-targeting liposomes should be explored.

We previously reported the novel concept of “dual-targeting” as an active targeting strategy, in which 2 different kinds of targeting peptides are used to modify drug-carrying liposomes [19]. In a previous study, we focused on the delivery of anticancer drugs to tumor angiogenic endothelial cells. Therefore, we used APRPG and GNGRG as ligands, since these peptides were isolated to have binding ability toward angiogenic vessels [10], [14]. The concept of “dual-targeting” was proved by using the combination of APRPG and GNGRG: “dual-targeting” not additively but synergistically enhanced the binding of liposomes modified with both ligands to activated HUVECs. However, it is still possible that the synergistic effect obtained was specific to this particular combination, APRPG and GNGRG. Therefore, in the present study, we examined a different combination of ligands, namely, APRPG and GRGDS, to prove the more general usefulness of dual-targeting.

At first, we examined the affinity of DT liposomes for proliferating HUVECs as an *in vitro* model of angiogenic endothelial cells. As a result, Dual-PEG-Lip remarkably showed higher affinity for the HUVECs compared with ST liposomes. This finding suggests that dual-targeting enhanced the association and transition of these liposomes to proliferating HUVECs. To clarify the mode of action for this cooperative interaction of ligands on the liposomal membrane, we next investigated whether DT liposomes had strong affinity for VEGFR-1 and α_v_β_3_ integrin by using SPR. APRPG or GRGDS peptide-decorated ST liposomes specifically bound to immobilized VEGFR-1 or α_v_β_3_ integrin, respectively. Interestingly, either of the ST liposomes bound more to the Biacore sensor chip covered with the corresponding target molecules than did the DT liposomes. This is a first report to show the binding of DT liposomes to the corresponding receptors (VEGFR-1 and integrin α_v_β_3_) at the molecular level.

Assuming that the surface area occupied by a DSPE-PEG molecule with or without peptide on a bilayer membrane is the same as that of DSPE, and that the PEG-conjugated lipids are evenly distributed in the outer and inner leaflets of liposomes, a single liposome with the size of 120 nm in diameter would be expected to expose about 6,600 PEG molecules or PEG-peptide molecules on its surface. Therefore, one PRP-PEG-decorated liposome would expose 6,600 APRPG molecules on its surface; and Dual-PEG-Lip, 3,300 APRPG and 3,300 GRGDS molecules. PRP-PEG-Lip, however, bound more than Dual-PEG-Lip to the VEGFR-1-immobilized chip: The difference in resonance units from the control for PRP-PEG-Lip was almost twice as much as that for Dual-PEG-Lip. Similarly, RGD-PEG-Lip was bound more to the sensor chip coated with integrin α_v_β_3 _than Dual-PEG-Lip. A possible explanation for these results is that the density of peptides on the liposomal surface would be important factor for the binding and that 3,300 peptides on the liposomal surface was not enough for the maximum binding.

In contrast to the binding of ST liposomes to the sensor chip coated with both target molecules, the DT liposomes bound to the sensor chip to a notably higher extent. However, the synergism in the binding of both ligands seemed to be higher in the cellular system, namely, binding to HUVECs. We speculate that, in this cellular system, liposomal uptake into the cells occurred and that the stronger binding by dual-targeting enhanced this uptake. Alternatively, the target molecules might be more available to bind ligands due to the mobility of the target molecules on a fluid cellular membrane. However, we must address the density of target molecules and mode of binding before clarifying the mechanism of the synergistic effect of dual targeting.

We next examined the biodistribution of dual-targeting liposomes in Colon26 NL-17-bearing mice. The result suggested that Dual-PEG-Lip accumulated in the tumor tissue significantly more than the other types of liposomes. RGD-PEG-Lip accumulated in the spleen. A previous study also showed that liposomes modified with RGD accumulate in the spleen after intravenous injection [9]. Because maybe the splenic macrophage has integrin on its surface [9], it is targeted by the RGD peptide motif. Similarly, we previously observed that GNGRG-decorated liposomes tend to accumulate in the spleen but that DT liposomes with APRPG ligands do not [19]. Consequently, Dual-PEG-Lip remained in the plasma more than RGD-PEG-Lip. This finding might be one of utility for dual-targeting.

Concerning the intratumoral distribution of DT liposomes, Dual-PEG-Lip mostly accumulated on angiogenic vessels. Furthermore, Dual-Lip also accumulated in the peripheral area around these vessels. This result might have been due to the interaction between GRGDS peptide and integrin α_v_β_3_ which is expressed on the tumor cell surface. This might be another advantage of dual-targeting. Taken together, our findings indicate that dual-targeting strategy would be useful for active targeting DDS.

### Conclusions

In the present study, the concept of dual-targeting was proved by using another combination of ligands to generalize the usefulness of the dual-targeting strategy. We clarified the usefulness of dual-targeting of tumor angiogenic vessels by use of VEGFR-1-targeted peptide APRPG and integrin α_v_β_3_-targeted peptide GRGDS. Liposomes decorated with both peptides synergistically associated with proliferative HUVECs, and also highly bound to immobilized target molecules as measured by SPR. Corresponding to this characteristic of dual-targeting liposomes, they also highly accumulated in tumor tissues of tumor-bearing mice. Moreover, the intratumoral distribution study indicated that the dual-targeting liposomes not only became localized on angiogenic vessels but also were distributed around these vessels. We speculate that GRGDS interacted with both angiogenic endothelial cells and tumor cells, although further experiments are needed to clarify this cooperative function of dual-targeting liposomes. In conclusion, dual-targeting would enhance the targeting ability of drug carriers in DDS.
